# Altered Cord Blood Enzyme Activities in Preeclampsia Without Severe Features: A Comparative Observational Study

**DOI:** 10.3390/medicina62101930

**Published:** 2026-10-07

**Authors:** Esra Şentürk, Elmin Eminov

**Affiliations:** 1 Department of Physiology, Faculty of Medicine, Ağrı İbrahim Çeçen University, 04100 Ağrı, Türkiye; 2 Department of Gynecology and Obstetrics, Faculty of Medicine, Düzce University, 81100 Düzce, Türkiye

**Keywords:** preeclampsia, umbilical cord blood, acetylcholinesterase, butyrylcholinesterase, carbonic anhydrase, glucose-6-phosphate dehydrogenase, glutathione reductase, oxidative stress

## Abstract

*Background and Objectives*: Preeclampsia (PE) is associated with placental dysfunction, endothelial abnormalities, and disturbed redox homeostasis, but enzyme activity patterns in the fetal circulation remain incompletely characterized. This study aimed to compare selected cord blood enzyme activities between pregnancies complicated by PE without severe features and normotensive pregnancies. *Materials and Methods*: This prospective observational comparative study included 122 participants: 54 women with PE without severe features and 68 women with normotensive pregnancies. All participants delivered vaginally at 37 weeks 1 day to 38 weeks 0 days of gestation. Plasma butyrylcholinesterase (BChE) and erythrocyte acetylcholinesterase (AChE), carbonic anhydrase (CA), glucose-6-phosphate dehydrogenase (G6PD), and glutathione reductase (GR) activities were measured spectrophotometrically. Groups were compared using the two-sided Mann–Whitney U test. *Results*: Compared with the normotensive pregnancy group, the PE group had lower median AChE (17.60 vs. 26.49 U/g Hb), BChE (1.060 vs. 1.340 EU/mg protein), G6PD (13.79 vs. 15.04 U/g Hb), and GR activities (0.880 vs. 1.075 EU/mg protein), corresponding to reductions of 33.6%, 20.9%, 8.3%, and 18.1%, respectively. Median CA activity was 3.1% higher in the PE group (8.615 vs. 8.360 EU/mg protein). All between-group differences were statistically significant (all *p* < 0.001). *Conclusions*: PE without severe features was associated with an enzyme-specific cord blood profile characterized by reduced cholinesterase and redox-related enzyme activities and increased CA activity. These findings support further investigation of fetal enzymatic alterations in PE.

## 1. Introduction

Preeclampsia (PE) is a complex multisystem disorder of pregnancy characterized by new-onset hypertension after 20 weeks of gestation, accompanied by proteinuria or, in its absence, specified manifestations of maternal organ dysfunction [1]. Preeclampsia without severe features is characterized by new-onset hypertension with proteinuria and the absence of severe features [1]. It remains an important cause of maternal and perinatal morbidity and mortality worldwide and is associated with adverse outcomes, including fetal growth restriction, preterm birth, placental abruption, and maternal renal, hepatic, neurological, and cardiovascular complications. Although the clinical manifestations of PE are well established, its pathogenesis is heterogeneous and incompletely understood. Current evidence supports a model in which abnormal placentation and inadequate remodeling of the maternal spiral arteries result in impaired uteroplacental perfusion and placental hypoxia, followed by the release of anti-angiogenic, inflammatory, and vasoactive factors into the maternal circulation. These processes contribute to systemic endothelial dysfunction and the clinical manifestations of PE [2,3,4,5].

Oxidative stress is considered an important component of the pathophysiological processes associated with PE. Reduced uteroplacental perfusion and repeated hypoxic-reoxygenation events can increase reactive oxygen species (ROS) generation, while mitochondrial dysfunction and inflammatory activation may further disturb redox homeostasis. Excessive ROS can promote lipid, protein, and deoxyribonucleic acid (DNA) oxidation and interfere with nitric oxide bioavailability and endothelial signaling, thereby contributing to vascular dysfunction. However, oxidative stress in PE occurs within a broader network of interacting processes that includes placental hypoxia, inflammation, angiogenic imbalance, mitochondrial dysfunction, and maternal vascular responses rather than representing an isolated biochemical abnormality [5,6,7].

Several enzymatic systems contribute to cellular protection against redox imbalance. Glucose-6-phosphate dehydrogenase (G6PD) generates reduced nicotinamide adenine dinucleotide phosphate (NADPH) through the pentose phosphate pathway, providing reducing equivalents required for cellular antioxidant processes, while glutathione reductase (GR) uses NADPH to regenerate reduced glutathione from oxidized glutathione. Alterations in these enzymes may therefore influence the capacity of erythrocytes to maintain redox homeostasis under oxidative challenge. Previous studies have reported impaired erythrocyte redox regulation and decreased G6PD activity in pregnancies complicated by PE, including evidence of altered redox status in fetal endothelial cells [8]. However, findings from maternal and fetal blood studies have not been entirely consistent, with antioxidant enzyme activities showing different patterns between maternal and cord blood in pregnancies complicated by PE. For example, Suhail et al. reported decreased SOD activity in preeclamptic cord blood compared with paired maternal blood, whereas catalase activity was increased in cord blood, highlighting the compartment-specific nature of the fetal antioxidant response [9].

Beyond classical redox-related enzymes, cholinesterases may provide complementary information about the biochemical consequences of PE. Acetylcholinesterase (AChE) and butyrylcholinesterase (BChE) hydrolyze choline esters and are involved in cholinergic signaling, but their activities have also been associated with inflammatory, metabolic, and vascular processes. Altered cholinesterase activity has been reported in hypertensive pregnancy disorders. In particular, a recent meta-analysis of 15 studies involving more than 1100 pregnant women reported significantly lower circulating BChE activity in women with PE than in healthy pregnant controls, supporting an association between reduced BChE activity and the systemic physiological alterations accompanying PE [10]. Earlier clinical studies have also investigated cholinesterase activities in relation to preeclampsia severity. Kurdoğlu et al. reported lower BChE activity in women with severe PE compared with healthy controls, whereas AChE activity did not differ significantly among women with mild PE, severe PE, and healthy pregnancies [11]. These observations suggest that cholinesterases may reflect biochemical changes associated with PE beyond those captured by conventional oxidative stress markers.

Carbonic anhydrases (CAs) represent another physiologically relevant enzyme family because they catalyze the reversible hydration of carbon dioxide and participate in carbon dioxide transport and acid-base regulation. Certain CA isoforms, particularly carbonic anhydrase IX (CA IX), are responsive to cellular hypoxia and have attracted increasing attention in the context of placental dysfunction and preeclampsia. Recent studies have reported increased circulating CA IX levels in women with preeclampsia or in women who subsequently develop the disease, supporting a potential association between CA IX and placental hypoxia-related processes. Galbiati et al. reported an early increase in circulating CA IX concentrations preceding the clinical onset of preeclampsia, particularly from the 28th gestational week onward. More recently, Ozgen and Dincgez investigated CA IX in early-onset preeclampsia and reported its potential association with fetal growth restriction. However, these studies evaluated CA IX concentration rather than total erythrocyte carbonic anhydrase enzymatic activity. Therefore, the relevance of altered total erythrocyte CA activity in cord blood remains insufficiently characterized [12,13]. Although these studies have primarily evaluated circulating CA IX concentrations rather than total erythrocyte CA enzymatic activity, they provide a physiological rationale for investigating carbonic anhydrase activity in the fetal circulation of pregnancies complicated by PE.

The fetal circulation is of particular interest because the fetus develops within the altered intrauterine environment created by placental dysfunction. Umbilical cord blood provides an accessible biological matrix that reflects aspects of the fetal biochemical environment at delivery. Previous studies have demonstrated alterations in oxidative stress and antioxidant status in cord blood from neonates born to mothers with PE. These alterations have included increased lipid peroxidation and oxidative DNA or protein damage, together with changes in antioxidant enzymes and total antioxidant status [9,14,15]. Importantly, maternal and fetal blood do not necessarily exhibit identical biochemical responses. Suhail et al. reported differences in the patterns of malondialdehyde and antioxidant enzyme activities between maternal and cord blood, highlighting the possibility of compartment-specific fetal responses to the preeclamptic environment. Taken together, these findings suggest that the fetal biochemical response to PE may involve dynamic and enzyme-specific adaptations rather than a uniform impairment of antioxidant defense.

This distinction is important when interpreting enzyme activity changes in cord blood. Increased or decreased enzyme activity may reflect different biological processes, including compensatory responses, altered enzyme regulation, or impaired adaptive capacity, depending on the enzyme and biological context. Accordingly, simultaneous assessment of enzymes involved in different physiological pathways may provide a broader characterization of fetal biochemical responses than evaluation of a single oxidative stress marker or enzyme system.

Despite growing evidence for oxidative, inflammatory, metabolic, and vascular disturbances in PE, relatively few studies have simultaneously examined different classes of enzymes in umbilical cord blood. In particular, the combined assessment of cholinesterases, carbonic anhydrase, and redox-related enzymes may provide complementary information about the biochemical response of the fetal circulation to PE. Such an approach may also help distinguish between enzymes involved directly in antioxidant defense and those that may reflect broader physiological adaptations to the altered intrauterine environment.

Previous studies have investigated individual cholinesterase, antioxidant, and carbonic anhydrase-related markers in maternal or fetal samples from pregnancies complicated by PE. However, these studies have generally focused on individual enzymatic pathways or limited biomarker panels, and fetal responses have not necessarily paralleled maternal changes (Supplementary Table S1). Although cord-blood BChE activity has previously been investigated in PE, to our knowledge, no study identified in our literature search has simultaneously evaluated AChE, BChE, CA, G6PD, and GR activities in umbilical cord blood within the same PE cohort. This integrated approach may provide a broader characterization of enzyme-specific biochemical alterations associated with the preeclamptic intrauterine environment.

Therefore, the present study aimed to comparatively evaluate AChE, BChE, CA, G6PD, and GR activities in cord blood obtained from pregnancies complicated by PE without severe features and normotensive pregnancies. By examining these complementary enzymatic systems within the same study population, we sought to characterize the enzyme activity profile associated with PE without severe features and to explore whether the observed alterations may reflect changes in fetal redox homeostasis, cholinergic regulation, and metabolic adaptation to the PE-associated intrauterine environment.

## 2. Materials and Methods

### 2.1. Study Design and Participants

This prospective observational, comparative study was conducted at Ağrı İbrahim Çeçen University Application and Research Hospital, Ağrı, Türkiye. The study population consisted of pregnant women who delivered at the hospital and were classified into two groups according to the presence or absence of preeclampsia (PE): a PE without severe features group and a normotensive pregnancy (NP) group.

Cord blood samples were collected between May 2023 and June 2024. Inclusion criteria were delivery at the study hospital, residence in the same geographical region, vaginal delivery at 37 weeks 1 day to 38 weeks 0 days of gestation, and classification as either preeclampsia without severe features, confirmed by clinical findings documented in the hospital records, or normotensive pregnancy without a history or clinical evidence of hypertensive disorders of pregnancy. Neonatal sex and birth-weight data were not available for analysis.

According to ACOG Practice Bulletin No. 222, preeclampsia without severe features is defined by new-onset systolic blood pressure ≥ 140 mmHg and/or diastolic blood pressure ≥ 90 mmHg on two occasions at least 4 h apart after 20 weeks of gestation in a previously normotensive woman, together with proteinuria and the absence of severe features. Proteinuria is defined as ≥300 mg protein in a 24 h urine collection, a urine protein-to-creatinine ratio ≥ 0.3, or a dipstick reading of 2+ when quantitative methods are unavailable [1]. In the present study, preeclampsia was diagnosed by an obstetrician and gynecologist (Elmin Eminov, co-author) based on blood pressure measurements obtained after 20 weeks of gestation in women with previously normal blood pressure and clinical laboratory findings obtained using the hospital laboratory equipment. Clinical and laboratory findings were used to classify participants as having preeclampsia without severe features.

Exclusion criteria were preeclampsia with severe features, pre-existing hypertension, diabetes mellitus, renal disease, cardiovascular disease, autoimmune disease, chronic inflammatory disease, or other clinically significant systemic disorders.

All chemicals and reagents used in this study were purchased from Sigma-Aldrich Chemie GmbH (Taufkirchen, Germany).

### 2.2. Cord Blood Collection and Preparation of Blood Fractions

Immediately after delivery, approximately 2 mL of umbilical cord blood was collected from each participant into EDTA-containing tubes (Ayset Tıbbi Ürünler, Adana, Türkiye). Following collection, the samples were transported to the Central Research Laboratory of Ağrı İbrahim Çeçen University for processing.

During the pre-analytical period, samples were transported on ice and maintained at approximately 4 °C until processing. Enzyme analyses were completed on the same day as sample collection.

Blood samples were centrifuged at 3000× *g* for 10 min to separate the plasma and erythrocyte fractions. The plasma fraction was carefully collected and used for the determination of BChE activity.

The erythrocyte fraction was washed twice with 0.9% NaCl solution to remove residual plasma and extracellular components. The washed erythrocytes were then lysed by adding three volumes of ice-cold distilled water to one volume of erythrocyte suspension (1:3, *v*/*v*). Following hemolysis, the samples were centrifuged at 3500× *g* for 10 min using a ROTOFIX 32 A benchtop centrifuge (Andreas Hettich GmbH, Tuttlingen, Germany). The resulting hemolysate supernatants were used for the determination of AChE, CA, GR, and G6PD activities.

All enzyme activity measurements were performed in three technical replicates for each sample, and the mean value of the replicate measurements was used for statistical analysis.

### 2.3. Determination of Hemoglobin Concentration and Total Protein

Hemoglobin (Hb) concentration was obtained from the complete blood count analysis of the cord blood samples using a hematology analyzer (HORIBA ABX SAS, Montpellier, France). Hb values were used to normalize erythrocyte AChE and G6PD activities, which were expressed as units per gram of hemoglobin (U/g Hb).

Total protein concentration in plasma and erythrocyte hemolysate samples was determined using the Bradford method with bovine serum albumin (BSA) as the standard [16]. Absorbance was measured at 595 nm, and protein concentrations were calculated from the corresponding calibration curve.

Plasma BChE and erythrocyte CA and GR activities were normalized to total protein concentration and expressed as enzyme units per milligram of protein (EU/mg protein). One enzyme unit (EU) was defined as the amount of enzyme catalyzing the conversion of 1 µmol of substrate per minute under the specified assay conditions.

### 2.4. Determination of Butyrylcholinesterase Activity

Plasma butyrylcholinesterase (BChE) activity was determined using the spectrophotometric method described by Ellman et al. [17], with modifications for a microplate format. The assay was based on the hydrolysis of S-butyrylthiocholine chloride and the subsequent reaction of the released thiol groups with 5,5′-dithiobis(2-nitrobenzoic acid) (DTNB), producing a colored product.

The reaction mixture was prepared in a microplate at room temperature (approximately 25 °C) in a final volume of 350 µL. The reaction mixture consisted of 10 µL of plasma sample, 200 µL of phosphate buffer (1 M, pH 8.0), 50 µL of DTNB solution (0.5 mM), 40 µL of distilled water, and 50 µL of S-butyrylthiocholine chloride solution (10 mM).

The reaction was initiated by the addition of the plasma sample. The change in absorbance at 412 nm was monitored for 5 min using a Multiskan GO microplate reader (Thermo Scientific, Waltham, MA, USA). BChE activity was calculated from the rate of change in absorbance and normalized to total protein concentration. The results were expressed as EU/mg protein.

Each sample was analyzed in three technical replicates, and the mean activity was used for subsequent statistical analysis.

### 2.5. Determination of Acetylcholinesterase Activity

Erythrocyte acetylcholinesterase (AChE) activity was determined using the spectrophotometric method described by Ellman et al. [17], with modifications for a microplate format. The assay was based on the hydrolysis of acetylthiocholine iodide and the subsequent reaction of the released thiol groups with DTNB, producing a chromogenic product measurable at 412 nm.

The reaction mixture was prepared in a microplate at room temperature (approximately 25 °C) in a final volume of 350 µL. The reaction mixture consisted of 10 µL of erythrocyte hemolysate, 200 µL of Tris-HCl buffer (1 M, pH 8.0), 50 µL of DTNB solution (0.5 mM), 40 µL of distilled water, and 50 µL of acetylthiocholine iodide solution (10 mM).

The reaction was initiated by the addition of the erythrocyte hemolysate. The change in absorbance at 412 nm was monitored for 5 min using a Multiskan GO microplate reader (Thermo Scientific). AChE activity was calculated from the rate of change in absorbance and normalized to hemoglobin concentration. The results were expressed as U/g Hb.

Each sample was analyzed in three technical replicates, and the mean activity was used for subsequent statistical analysis.

### 2.6. Determination of Carbonic Anhydrase Activity

Erythrocyte carbonic anhydrase (CA) activity was determined spectrophotometrically according to the method described by Verpoorte et al. [18], with modifications for a microplate format. The assay was based on the hydrolysis of 4-nitrophenyl acetate (4-NPA) to 4-nitrophenolate, which was monitored by measuring the change in absorbance at 348 nm.

The reaction mixture was prepared in a microplate at room temperature (approximately 25 °C) in a final volume of 350 µL. The reaction mixture consisted of 180 µL of 0.05 M Tris-SO_4_ buffer (pH 7.4), 100 µL of 3 mM 4-NPA, 60 µL of distilled water, and 10 µL of erythrocyte hemolysate.

The reaction was monitored at 348 nm for 3 min using a Multiskan GO microplate reader. CA activity was calculated from the change in absorbance over the measurement period and normalized to total protein concentration. The results were expressed as EU/mg protein.

Each sample was analyzed in three technical replicates, and the mean activity was used for subsequent statistical analysis.

### 2.7. Determination of Glutathione Reductase Activity

Erythrocyte glutathione reductase (GR) activity was determined according to the method described by Beutler [19], with modifications for a microplate format. The assay was based on the NADPH-dependent reduction in oxidized glutathione (GSSG) to reduced glutathione (GSH), accompanied by oxidation of NADPH. The decrease in absorbance at 340 nm was monitored spectrophotometrically.

The assay system contained 100 mM Tris-HCl buffer (pH 8.0) supplemented with 0.5 mM EDTA, 3.3 mM GSSG, and 0.1 mM NADPH. The reaction mixture was prepared in a microplate in a final volume of 350 µL and contained 10 µL of erythrocyte hemolysate together with the assay components.

The reaction was initiated by the addition of the erythrocyte hemolysate, and the decrease in absorbance at 340 nm was recorded for 3 min using a Multiskan GO microplate reader (Thermo Scientific). GR activity was calculated from the rate of NADPH oxidation and normalized to total protein concentration. The results were expressed as EU/mg protein.

Each sample was analyzed in three technical replicates, and the mean activity was used for subsequent statistical analysis.

### 2.8. Determination of Glucose-6-Phosphate Dehydrogenase Activity

Erythrocyte glucose-6-phosphate dehydrogenase (G6PD) activity was determined according to the method described by Beutler [19], with modifications for a microplate format. The assay was based on the oxidation of glucose-6-phosphate (G6P) coupled with the reduction in NADP^+^ to NADPH in the presence of G6PD. The formation of NADPH was monitored spectrophotometrically at 340 nm.

The assay system contained 100 mM Tris-HCl buffer (pH 8.5), supplemented with 0.5 mM EDTA, 10 mM MgCl_2_, 0.2 mM NADP^+^, and 0.6 mM G6P. The reaction mixture was prepared in a microplate in a final volume of 350 µL and contained 10 µL of erythrocyte hemolysate together with the assay components.

The reaction was initiated by the addition of the erythrocyte hemolysate. The increase in absorbance at 340 nm was recorded for 3 min using a Multiskan GO microplate reader (Thermo Scientific). G6PD activity was calculated from the rate of NADPH formation and normalized to hemoglobin concentration. The results were expressed as U/g Hb.

Each sample was analyzed in three technical replicates, and the mean activity was used for subsequent statistical analysis.

### 2.9. Statistical Analysis

Statistical analyses were performed using GraphPad Prism version 9.0 (GraphPad Software, San Diego, CA, USA). The distribution of each enzyme activity variable within each study group was assessed using the Shapiro–Wilk test. Because the enzyme activity data did not consistently satisfy the assumption of normality, all enzyme activities were summarized as median and interquartile range (IQR), and comparisons between the PE and NP groups were performed using the two-sided Mann–Whitney U test. For enzyme activity measurements, the three technical replicate measurements obtained for each participant were averaged before statistical analysis; therefore, each participant contributed a single independent observation to the group comparison. A *p*-value <0.05 was considered statistically significant. Maternal age and gestational age at delivery were summarized as mean ± standard deviation and compared using two-sided Mann–Whitney U tests. Gestational age was calculated in completed days (259 plus the recorded days beyond 37 weeks). Additional analyses were performed using SciPy version 1.17.0. For each enzyme, robust Z-scores were calculated for both groups using the same normotensive reference: the participant value minus the NP median, divided by 1.4826 times the NP median absolute deviation (MAD). MAD was defined as the median absolute deviation from the group median. This standardization facilitated comparison across enzyme units without implying a normal distribution or diagnostic performance. Within-group associations between enzyme activities were evaluated using two-sided Spearman rank correlations. The Benjamini–Hochberg procedure controlled the false discovery rate separately across the 10 enzyme-pair correlations in each group; q < 0.05 was considered significant for these exploratory analyses. Correlation *p*-values used the asymptotic approximation. Group comparisons were unadjusted for covariates.

### 2.10. Ethical Considerations

The study was conducted in accordance with the principles of the Declaration of Helsinki. Ethical approval was obtained from the Scientific Ethics Committee of Atatürk University (Approval No. B.30.2.ATA.0.01.00/192). Written informed consent was obtained from all participants before inclusion in the study.

All biological samples and participant information were handled confidentially and were used exclusively for the purposes approved by the relevant ethics committee.

## 3. Results

A total of 122 participants were included in the analysis, comprising 68 women in the normotensive pregnancy (NP) group and 54 women in the preeclampsia without severe features (PE) group. Mean maternal age was 28.60 ± 2.84 years in the NP group and 28.20 ± 2.15 years in the PE group (*p* = 0.681), with an overall range of 25–37 years. Mean gestational age at delivery was 261.54 ± 1.08 days in the NP group and 262.72 ± 1.69 days in the PE group (*p* < 0.001). All participants delivered vaginally; gestational age ranged from 37 weeks 1 day to 37 weeks 5 days in the NP group and from 37 weeks 1 day to 38 weeks 0 days in the PE group. The maternal and obstetric characteristics of the study groups are summarized in Table 1.

### 3.1. Acetylcholinesterase Activity

Erythrocyte acetylcholinesterase (AChE) activity was significantly lower in the PE group than in the NP group. The median AChE activity was 26.49 (25.08–27.55) U/g Hb in the NP group and 17.60 (16.98–18.42) U/g Hb in the PE group. Thus, median AChE activity was approximately 33.6% lower in cord blood from neonates born to mothers with PE without severe features (Mann–Whitney U = 33.5, *p* < 0.001).

### 3.2. Butyrylcholinesterase Activity

Plasma butyrylcholinesterase (BChE) activity was also significantly lower in the PE group. The median BChE activity was 1.340 (1.280–1.380) EU/mg protein in the NP group and 1.060 (0.980–1.118) EU/mg protein in the PE group. This corresponded to an approximately 20.9% reduction in median BChE activity in the PE group (Mann–Whitney U = 126.0, *p* < 0.001).

### 3.3. Carbonic Anhydrase Activity

In contrast to the cholinesterase activities, erythrocyte carbonic anhydrase (CA) activity was significantly higher in the PE group. The median CA activity was 8.360 (7.860–8.620) EU/mg protein in the NP group and 8.615 (8.325–8.928) EU/mg protein in the PE group, corresponding to an approximately 3.1% increase in the PE group (Mann–Whitney U = 1098.5, *p* < 0.001).

### 3.4. Glucose-6-Phosphate Dehydrogenase Activity

Erythrocyte glucose-6-phosphate dehydrogenase (G6PD) activity was significantly lower in the PE group than in the NP group. The median G6PD activity was 15.04 (14.24–15.44) U/g Hb in the NP group and 13.79 (12.96–14.08) U/g Hb in the PE group. This represented an approximately 8.3% reduction in median G6PD activity in the PE group (Mann–Whitney U = 395.5, *p* < 0.001).

### 3.5. Glutathione Reductase Activity

Similarly, erythrocyte glutathione reductase (GR) activity was significantly lower in cord blood from neonates born to mothers with PE without severe features. The median GR activity was 1.075 (1.010–1.110) EU/mg protein in the NP group and 0.880 (0.860–0.910) EU/mg protein in the PE group. Median GR activity was approximately 18.1% lower in the PE group than in the NP group (Mann–Whitney U = 143.0, *p* < 0.001).

### 3.6. Overall Enzyme Activity Profile

**Figure 1:**
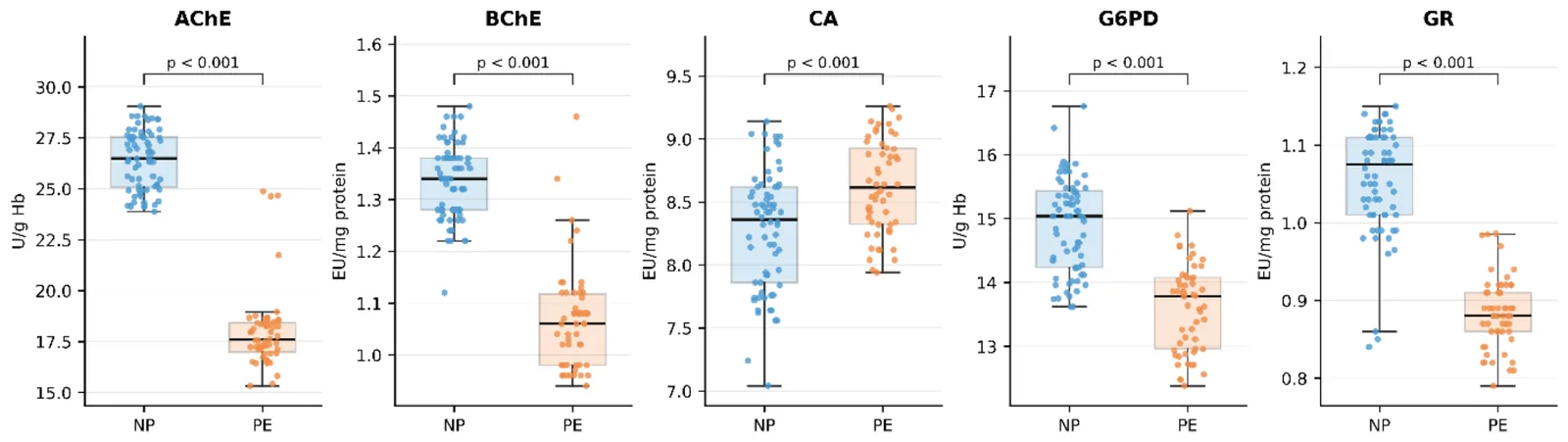
Distributions of cord blood enzyme activities in normotensive pregnancies (NP) and pregnancies complicated by preeclampsia (PE). Boxes represent the interquartile range, horizontal lines within the boxes indicate the median, and whiskers extend to the most extreme observations within 1.5 times the interquartile range. Individual dots represent individual participants. Between-group comparisons were performed using the two-sided Mann–Whitney U test; all comparisons were statistically significant (*p* < 0.001). AChE, acetylcholinesterase; BChE, butyrylcholinesterase; CA, carbonic anhydrase; G6PD, glucose-6-phosphate dehydrogenase; GR, glutathione reductase; Hb, hemoglobin.

Overall, the cord blood enzyme activity profile differed markedly between the PE and NP groups. Cord blood samples from neonates born to mothers with PE without severe features showed significantly lower AChE, BChE, G6PD, and GR activities, whereas CA activity was significantly higher. Among the investigated enzymes, the largest relative difference was observed for AChE, with a 33.6% lower median activity in the PE group, followed by BChE (20.9% lower), GR (18.1% lower), G6PD (8.3% lower), and CA (3.1% higher). The complete comparison of enzyme activities is presented in Table 2, and their distributions are shown in Figure 1.

### 3.7. Robust Standardized Enzyme Activity Profile

**Figure 2:**
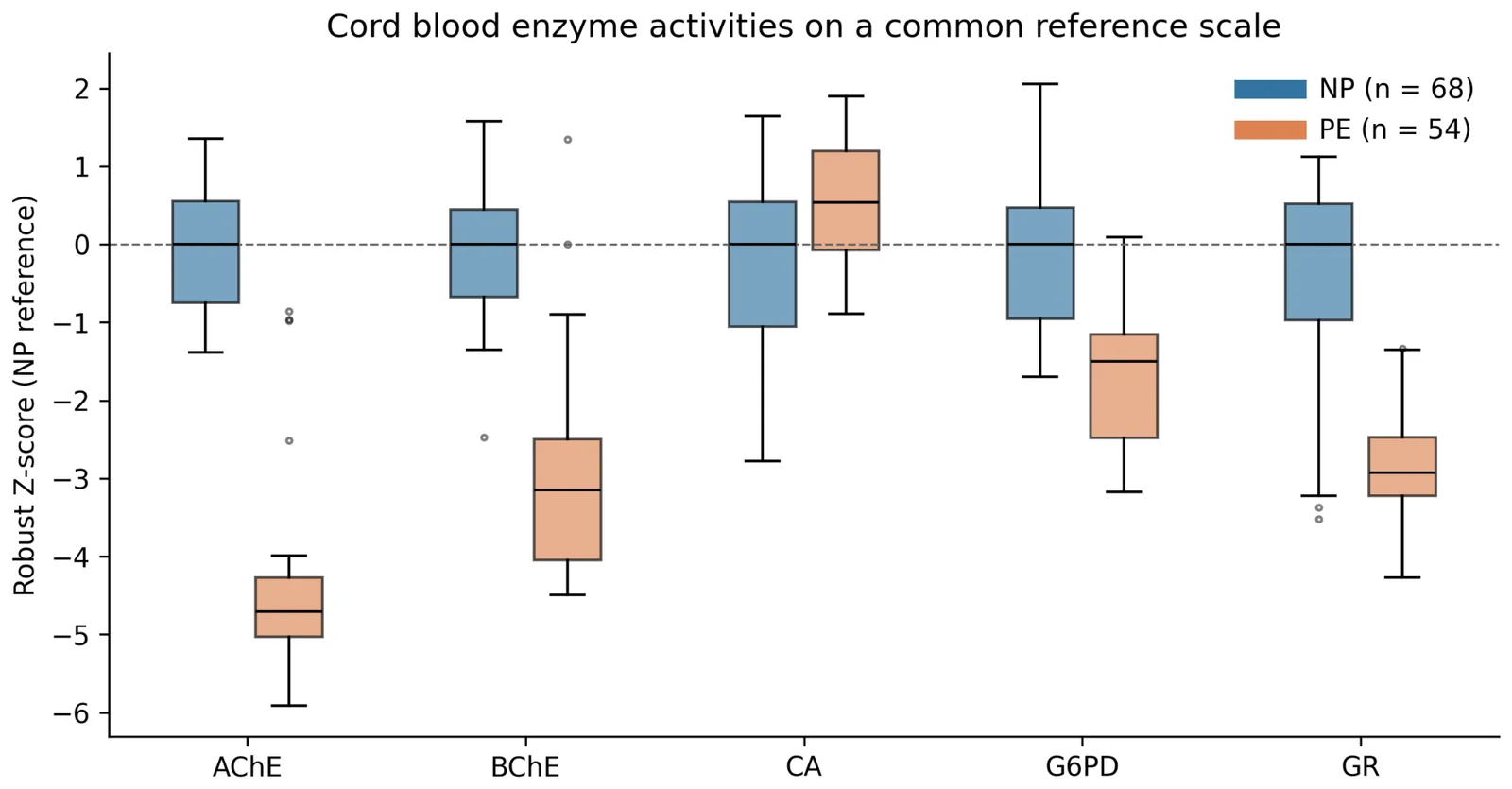
Robust Z-scores of cord blood enzyme activities. Both groups were standardized using each enzyme’s NP median and 1.4826 × NP MAD. Boxes show the interquartile range, lines indicate medians, whiskers extend to observations within 1.5 × IQR, and points show outliers. The dashed line denotes the NP reference median. NP, n = 68; PE, n = 54. Standardization preserves the ordering of observations and does not change the Mann–Whitney group comparisons.

Using the NP group as the common reference, median robust Z-scores in the PE group were −4.71 for AChE, −3.15 for BChE, −2.92 for GR, −1.50 for G6PD, and +0.54 for CA. AChE showed the largest absolute standardized median difference from the NP reference. These values describe relative shifts on a common scale and do not establish diagnostic superiority. Distributions are shown in Figure 2; reference parameters and interquartile ranges are provided in Supplementary Table S2.

### 3.8. Within-Group Correlations

**Figure 3:**
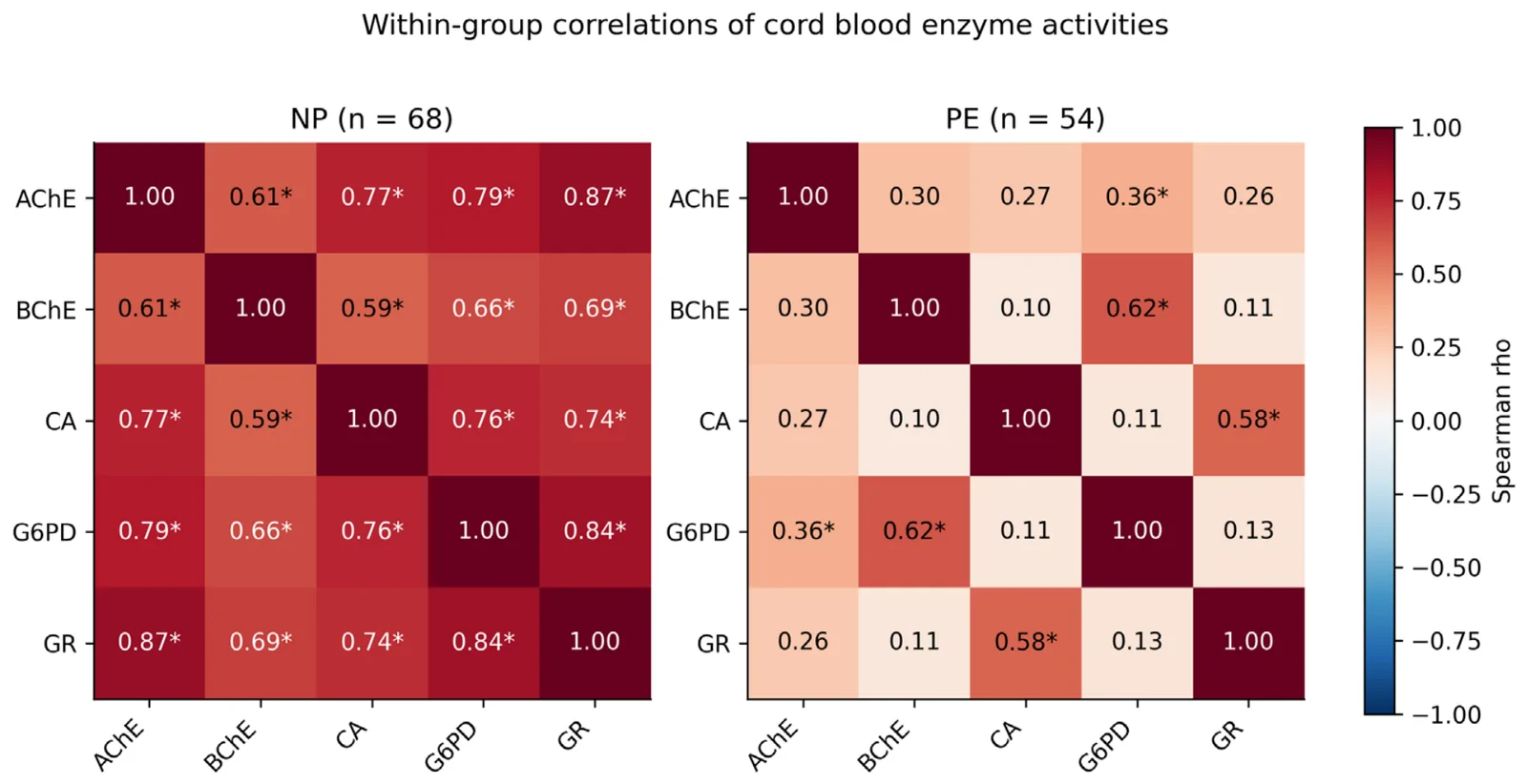
Within-group Spearman correlation matrices for the five enzyme activities. Cells show Spearman rho. Asterisks indicate q < 0.05 after Benjamini–Hochberg adjustment across 10 enzyme-pair tests within each group; diagonal entries represent self-correlations and were not tested. NP, n = 68; PE, n = 54.

All 10 enzyme-pair correlations in the NP group were positive and significant after false discovery rate adjustment (rho = 0.592–0.873; all q < 0.001). In the PE group, three pairs remained significant: BChE–G6PD (rho = 0.618, q < 0.001), CA–GR (rho = 0.585, q < 0.001), and AChE–G6PD (rho = 0.362, q = 0.024). The remaining PE correlations did not meet the adjusted significance threshold. Figure 3 summarizes these exploratory associations; full coefficients, *p*-values, and q-values are provided in Supplementary Table S3. Correlation strengths were not formally compared between groups.

## 4. Discussion

The present study demonstrated a distinct alteration in the cord blood enzyme activity profile of neonates born to mothers with preeclampsia without severe features. Compared with normotensive pregnancies, cord blood samples in the preeclampsia group showed significantly lower AChE, BChE, G6PD, and GR activities, whereas CA activity was significantly higher. The magnitude of the observed differences varied among the enzymes, with the largest reduction observed for AChE (33.6%), followed by BChE (20.9%), GR (18.1%), and G6PD (8.3%). CA activity showed a comparatively modest increase of 3.1%. Taken together, these findings indicate that preeclampsia is associated with a differential rather than uniform alteration of enzymatic homeostasis in the fetal circulation.

The reduction in AChE and BChE activities observed in the present study suggests that cholinesterase-related pathways may be altered in the intrauterine environment associated with preeclampsia. Previous studies have reported alterations in cholinesterase activity in women with preeclampsia, although the findings have been more consistent for BChE than for AChE. Importantly, Kharb et al. reported significantly lower cord-blood BChE activity in pregnancies complicated by PE than in normotensive pregnancies [20]. A meta-analysis of 15 studies also demonstrated significantly lower circulating BChE activity in women with PE than in healthy pregnant controls [10]. Similarly, Rahimi et al. reported lower maternal BChE activity in preeclamptic women and demonstrated associations between BChE activity, oxidative stress, and lipid-related parameters [21]. Therefore, our finding of a 20.9% lower median cord-blood BChE activity in the PE group is consistent with previous maternal and cord-blood findings and provides further evidence of altered cholinesterase activity in the fetal circulation.

The AChE finding requires a more cautious interpretation because previous clinical studies have not consistently demonstrated a reduction in AChE activity in preeclampsia. Kurdoğlu et al., for example, reported differences in cholinesterase-related parameters according to preeclampsia severity but did not observe a significant difference in AChE among their study groups [11]. In contrast, the present study demonstrated a pronounced 33.6% reduction in median erythrocyte AChE activity in cord blood. This discrepancy may reflect differences in the biological compartment examined, the timing of sampling, gestational age, disease severity, or the characteristics of the study populations. Because AChE was measured in erythrocytes in the present study, the finding may reflect alterations in fetal erythrocyte cholinergic or membrane-associated physiology rather than changes in maternal circulating AChE alone.

The redox-related findings provide additional evidence of an altered biochemical environment in the fetal circulation. G6PD and GR are functionally linked components of cellular redox regulation. G6PD generates NADPH through the pentose phosphate pathway, whereas GR uses NADPH to regenerate reduced glutathione from oxidized glutathione. Therefore, reductions in both enzymes may be relevant to the capacity of erythrocytes to maintain reducing conditions under oxidative challenge. In the present study, median G6PD activity was 8.3% lower and median GR activity was 18.1% lower in the PE group than in the NP group. Although the magnitude of the G6PD reduction was relatively modest compared with the changes observed for AChE, BChE, and GR, the difference remained statistically significant.

The finding of reduced G6PD activity in the present study is compatible with previous evidence linking preeclampsia to altered erythrocyte redox regulation. Afzal-Ahmed et al. reported reduced G6PD activity in erythrocytes from women with preeclampsia, together with impaired redox regulation in erythrocytes and fetal endothelial cells [8]. However, findings have not been entirely consistent across maternal studies. Kahraman et al. reported no significant difference in erythrocyte G6PD activity between women with preeclampsia and normotensive pregnant women [22]. Thus, the lower G6PD activity observed in our cord blood samples may represent a fetal or compartment-specific alteration, although further maternal–fetal paired studies are required to determine whether this difference reflects a distinct fetal response to preeclampsia.

The reduction in GR activity observed in the present study is also relevant to the oxidative stress framework of preeclampsia. Previous studies have demonstrated alterations in antioxidant enzyme systems in maternal and cord blood in pregnancies complicated by preeclampsia, but the direction of change has not been uniform across biological compartments or enzymes. Suhail et al. reported decreased maternal antioxidant enzyme activities in preeclampsia, whereas cord blood did not simply reproduce the maternal pattern and showed evidence of preserved or compensatory antioxidant responses [9]. These observations emphasize that maternal and fetal redox responses should not be assumed to be identical. The simultaneous reduction in G6PD and GR in our cord blood samples may nevertheless suggest a reduced capacity of fetal erythrocytes to maintain NADPH-dependent glutathione recycling under the conditions associated with preeclampsia.

An important finding of the present study was the significantly higher erythrocyte CA activity in the PE group. Although the relative difference was modest (3.1%), the between-group comparison was statistically significant. CAs participate in carbon dioxide hydration, bicarbonate formation, and acid-base regulation, and certain CA isoforms are responsive to cellular hypoxia. Previous research in preeclampsia has particularly focused on CA IX expression or circulating concentrations as potential indicators of placental hypoxia. For example, Galbiati et al. reported increased circulating CA IX levels before the clinical onset of PE, supporting its potential association with hypoxia-related processes in preeclampsia [12]. More recently, Ozgen and Dincgez investigated CA IX in early-onset PE and its relationship with fetal growth restriction, further supporting the relevance of the CA IX-hypoxia axis in pregnancies affected by PE [13]. However, these studies evaluated CA IX rather than total erythrocyte CA enzymatic activity. Therefore, the higher CA activity observed in the present study may indicate an alteration in carbon dioxide/acid-base handling or a broader enzymatic adaptation of the fetal erythrocyte compartment in PE. Nevertheless, this finding should be interpreted cautiously, as the present assay measured total erythrocyte CA activity and did not distinguish among individual CA isoforms; therefore, it cannot be interpreted as direct evidence of increased CA IX expression.

The combined pattern observed in this study is noteworthy. AChE, BChE, G6PD, and GR activities were all lower in the PE group, whereas CA activity was higher. This pattern does not support a simple generalized increase or decrease in enzyme activity. Instead, it suggests that different enzymatic systems may respond differently to the altered intrauterine environment associated with preeclampsia. The reductions in G6PD and GR may be related to altered redox regulation, while the changes in cholinesterases may reflect alterations in cholinergic, inflammatory, metabolic, or membrane-associated processes. The increase in CA activity may represent a different adaptive response associated with carbon dioxide handling or hypoxia-related physiology.

The robust standardized profile placed the largest absolute median shift at AChE, consistent with its larger relative reduction. Correlation analyses also identified positive associations across the enzyme panel in the NP group and a smaller subset of associations meeting the adjusted significance threshold in the PE group. These exploratory findings may inform future investigation of joint enzyme activity patterns, but differences in significance do not demonstrate that correlation strengths differ between groups. Biological coupling cannot be inferred from correlations alone, particularly because several activities share hemoglobin or total-protein normalization denominators.

### Strengths and Limitations

Several strengths and limitations should be considered when interpreting these findings. A strength of the present study is the simultaneous assessment of five enzymatic systems in cord blood obtained from a relatively homogeneous population. All participants delivered vaginally within a narrow gestational-age interval. However, gestational age at delivery differed between groups by approximately 1.18 days on average. The enzyme activity comparisons were unadjusted for gestational age and maternal age; therefore, residual confounding cannot be excluded. Nevertheless, this study has several limitations. First, enzyme activities were measured in cord blood collected immediately after delivery; therefore, the findings provide only a cross-sectional snapshot of the fetal biochemical environment and cannot establish causality or determine whether the observed alterations preceded preeclampsia. Second, the study included only women with preeclampsia without severe features; consequently, the findings may not be generalizable to severe or early-onset preeclampsia. Third, neonatal sex and birth weight were not available and could not be evaluated as potential confounding variables. Fourth, maternal and cord blood samples were not evaluated as paired samples, and placental tissue was not examined. Fifth, the exploratory correlation analyses do not establish functional coupling or differences in correlation strength between groups; no formal between-group correlation comparison was performed. Finally, the CA assay measured total erythrocyte CA activity and did not distinguish individual CA isoforms; therefore, the higher CA activity cannot be interpreted as direct evidence of increased CA IX expression.

Despite these limitations, the present study provides a comprehensive comparison of five enzymatic systems within the same cord blood study population. The simultaneous assessment of cholinesterase, carbonic anhydrase, and redox-related enzymes allowed the identification of an enzyme-specific alteration in the fetal biochemical profile associated with preeclampsia. Further studies incorporating maternal-cord paired samples, placental tissue, disease severity, gestational age, neonatal outcomes, and additional oxidative stress markers may help determine whether these enzyme alterations represent compensatory adaptations, impaired defenses, or both.

## 5. Conclusions

Preeclampsia without severe features was associated with lower cord blood AChE, BChE, G6PD, and GR activities and higher CA activity compared with normotensive pregnancies. These findings indicate enzyme-specific alterations in the fetal blood compartment at delivery. Further studies are needed to establish their biological significance and potential clinical relevance.

## Figures and Tables

**Table 1:** Maternal and obstetric characteristics of the study groups.

Characteristic	Normotensive Pregnancy (n = 68) (n = 68)	Preeclampsia (n = 54) (n = 54)	*p*-Value
Maternal age, years	28.60 ± 2.84	28.20 ± 2.15	0.681
Gestational age at delivery, days	261.54 ± 1.08	262.72 ± 1.69	<0.001

Values are presented as mean ± standard deviation. Normotensive pregnancy (n = 68); Preeclampsia without severe features (n = 54); n, number of participants. Gestational age was expressed in completed days. *p*-values are from two-sided Mann–Whitney U tests. All participants delivered vaginally; delivery mode and PE classification are described in the text because they were fixed characteristics of the study population.

**Table 2:** Comparison of cord blood enzyme activities between normotensive and pregnancies complicated by preeclampsia without severe features.

Enzyme Activity	Normotensive Pregnancy (n = 68)	Preeclampsia (n = 54)	Change (%)	Mann–Whitney U	*p*-Value
AChE (U/g Hb)	26.49 (25.08–27.55)	17.60 (16.98–18.42)	−33.6	33.5	<0.001
BChE (EU/mg protein)	1.340 (1.280–1.380)	1.060 (0.980–1.118)	−20.9	126.0	<0.001
CA (EU/mg protein)	8.360 (7.860–8.620)	8.615 (8.325–8.928)	+3.1	1098.5	<0.001
G6PD (U/g Hb)	15.04 (14.24–15.44)	13.79 (12.96–14.08)	−8.3	395.5	<0.001
GR (EU/mg protein)	1.075 (1.010–1.110)	0.880 (0.860–0.910)	−18.1	143.0	<0.001

Values are presented as median (interquartile range). Percentage change was calculated as [(PE median − NP median)/NP median] × 100. Between-group comparisons were performed using the two-sided Mann–Whitney U test. AChE, acetylcholinesterase; BChE, butyrylcholinesterase; CA, carbonic anhydrase; G6PD, glucose-6-phosphate dehydrogenase; GR, glutathione reductase; Hb, hemoglobin; NP, normotensive pregnancy; PE, preeclampsia.

## Data Availability

The data supporting the findings of this study are available from the corresponding author upon reasonable request, subject to ethical and privacy restrictions.

## Supplementary Materials

The following supporting information can be downloaded at https://www.mdpi.com/article/10.3390/medicina62101930/s1: Table S1, Summary of previous studies evaluating selected enzymatic and redox-related markers in preeclampsia and comparison with the present study. Table S2, Normotensive reference parameters and robust Z-score summaries; Table S3, Within-group Spearman correlations with false discovery rate adjustment.
