# Supplementary material for: Altered Cord Blood Enzyme Activities in Preeclampsia Without Severe Features: A Comparative Observational Study
Source: Medicina (Kaunas). 2026 Oct 7;62(10):1930. doi: 10.3390/medicina62101930 (PMC13707027; doi:10.3390/medicina62101930)
Supplement: Supplementary file 1 [file medicina-62-01930-s001.zip › medicina-4569905-supplementary.pdf]

Supplementary Table S1. Summary of previous studies evaluating selected enzymatic and redox-related markers in preeclampsia and comparison with the present study

Enzyme/marker	Biological compartment	Study population/design	Main finding relevant to PE	Relevance to the present study	Representative reference(s)
**BChE**	Maternal and umbilical cord blood	25 preeclamptic pregnancies, 25 normotensive pregnancies, and 25 healthy non-pregnant controls	Cord-blood BChE activity was significantly lower in preeclamptic pregnancies than in normotensive pregnancies. Maternal BChE activity was also lower in PE.	Provides direct previous evidence of reduced cord-blood BChE activity in PE; however, erythrocyte AChE, CA, G6PD, and GR activities and the combined five-enzyme profile evaluated in the present study were not assessed.	[20]
**AChE**	Maternal whole blood	31 women with mild PE, 30 women with severe PE, and 50 healthy pregnant controls	Whole-blood AChE activity did not differ significantly among the mild PE, severe PE, and healthy pregnancy groups.	Contrasts with the pronounced reduction in erythrocyte AChE activity observed in cord blood in the present study and highlights the potential importance of the biological compartment examined.	[11]
**BChE**	Maternal circulation	Clinical studies and a systematic review/meta-analysis	Maternal circulating BChE activity was generally lower in women with PE than in normotensive pregnant controls.	Supports the direction of the present BChE finding and enables comparison with both maternal and previous cord-blood observations.	[10, 11, 21]
**G6PD**	Maternal erythrocytes and fetal endothelial cells	Women with normotensive or preeclamptic pregnancies	Reduced G6PD activity and impaired redox regulation were reported in erythrocytes and fetal endothelial cells from preeclamptic pregnancies, although findings have not been uniform across studies.	Provides biological support for investigating G6PD in PE while emphasizing possible differences between maternal and fetal compartments.	[8, 22]
**Other antioxidant enzymes (SOD, GPx, and catalase)**	Maternal and cord blood	Preeclamptic and normotensive pregnancies	Cord-blood antioxidant responses differed from maternal responses, with enzyme-specific changes involving SOD, GPx, and catalase as well as evidence of oxidative damage.	Supports the concept that fetal redox responses to PE may be heterogeneous rather than uniformly increased or decreased.	[9, 2, 15]
**Carbonic anhydrase-related marker (CA IX)**	Maternal circulation	Studies of PE, placental hypoxia-related processes, and fetal growth restriction	Increased circulating CA IX concentrations have been associated with PE and investigated in relation to placental hypoxia-related processes and fetal growth restriction.	Provides a physiological rationale for investigating CA in PE; however, previous studies evaluated CA IX concentration rather than total erythrocyte CA enzymatic activity.	[12, 13]
**AChE, BChE, CA, G6PD, and GR**	Umbilical cord blood	Present study: 68 normotensive and 54 PE pregnancies	AChE, BChE, G6PD, and GR activities were lower, whereas CA activity was higher in PE.	To our knowledge, no study identified in our literature search has simultaneously evaluated these five enzymatic activities within the same cord-blood PE cohort.	Present study

Abbreviations: AChE, acetylcholinesterase; BChE, butyrylcholinesterase; CA, carbonic anhydrase; CA IX, carbonic anhydrase IX; G6PD, glucose-6-phosphate dehydrogenase; GR, glutathione reductase; PE, preeclampsia; SOD, superoxide dismutase; GPx, glutathione peroxidase. Reference numbers correspond to the reference list of the main manuscript.

Table S2. Normotensive reference parameters and robust Z-score summaries

Enzyme	NP median	NP MAD	NP robust Z-score	PE robust Z-score
AChE	26.490	1.275	0.00 (-0.75 to 0.56)	-4.71 (-5.03 to -4.27)
BChE	1.340	0.060	0.00 (-0.67 to 0.45)	-3.15 (-4.05 to -2.50)
CA	8.360	0.320	0.00 (-1.05 to 0.55)	0.54 (-0.07 to 1.20)
G6PD	15.040	0.565	0.00 (-0.96 to 0.47)	-1.50 (-2.48 to -1.15)
GR	1.075	0.045	0.00 (-0.97 to 0.52)	-2.92 (-3.22 to -2.47)

Z-scores are shown as median (25th to 75th percentile). Both groups use the same NP reference. Robust Z-score = (participant activity − NP median)/(1.4826 × NP MAD). MAD is the median absolute deviation from the NP median. Reference median and MAD retain the original enzyme units: U/g Hb for AChE and G6PD; EU/mg protein for BChE, CA, and GR. Z-scores are dimensionless. The conversion does not imply normality or establish diagnostic accuracy.

Table S3. Within-group Spearman correlations

Group	Enzyme pair	n	Spearman rho	p-value	FDR q-value
NP	AChE–BChE	68	0.613	2.71e-08	3.01e-08
NP	AChE–CA	68	0.767	2.28e-14	5.71e-14
NP	AChE–G6PD	68	0.794	6.89e-16	2.3e-15
NP	AChE–GR	68	0.873	2.81e-22	2.81e-21
NP	BChE–CA	68	0.592	1.07e-07	1.07e-07
NP	BChE–G6PD	68	0.658	1.06e-09	1.32e-09
NP	BChE–GR	68	0.693	5.95e-11	8.5e-11
NP	CA–G6PD	68	0.761	5.03e-14	1.01e-13
NP	CA–GR	68	0.742	4.67e-13	7.78e-13
NP	G6PD–GR	68	0.838	5.31e-19	2.65e-18
PE	AChE–BChE	54	0.304	0.0253	0.0633
PE	AChE–CA	54	0.270	0.0481	0.0962
PE	AChE–G6PD	54	0.362	0.00715	0.0238
PE	AChE–GR	54	0.259	0.059	0.0983
PE	BChE–CA	54	0.098	0.48	0.48
PE	BChE–G6PD	54	0.618	6.28e-07	6.28e-06
PE	BChE–GR	54	0.111	0.423	0.473
PE	CA–G6PD	54	0.111	0.425	0.473
PE	CA–GR	54	0.585	3.45e-06	1.73e-05
PE	G6PD–GR	54	0.128	0.358	0.473

Two-sided Spearman correlations were calculated separately in each group using one participant-level value per enzyme. P-values use the asymptotic approximation. Benjamini–Hochberg false discovery rate adjustment was applied separately to the 10 tests in each group. q < 0.05 defines the exploratory significance threshold. All 10 NP pairs and three PE pairs (AChE–G6PD, BChE–G6PD, CA–GR) met this threshold. No formal between-group test of correlation coefficients was performed. Shared normalization denominators may contribute to observed associations.
